# Health, social, and dental professionals’ experiences of working within an extended home-visit program in the child healthcare: a qualitative interview study in Sweden

**DOI:** 10.1186/s12913-023-09791-z

**Published:** 2023-07-31

**Authors:** Elisabeth Mangrio, Maria Hjortsjö

**Affiliations:** 1 Department of Care Science, Malmö University, Malmö, Sweden; 2 Department of Social Work, Malmö University, Malmö, Sweden

**Keywords:** Child healthcare, Home visits, Interprofessional collaboration, Qualitative research

## Abstract

**Background:**

The goal of the Swedish child healthcare system is to reach all children with health-promotive actions and to create equal health opportunities for all children. In that context, an extended home-visit program – called Grow Safely – for first-time parents, with an interprofessional collaboration between child healthcare nurses, midwives, social workers, and dental assistants, was initiated. The current study aims at illuminating and evaluating the health, social, and dental professionals’ experiences of working within this program and how such collaboration could benefit the professions.

**Methods:**

A qualitative method was chosen, and 13 interviews were carried out with professionals working within child healthcare centers that participated in an extended home-visit program in the southernmost part of Sweden. The interviews were analyzed via Burnard’s approach to content analysis.

**Results:**

The results showed that it was satisfying for the health, social, and dental professionals to work with the home-visit program and that they encountered positive feelings among the parents receiving it. The creation of deep conversations and parents opening up about feelings that could otherwise be shameful to express, was a positive aspect of the home visits. A negative aspect was the difficulty of handling the (sometimes necessary) interpretation over the phone during the visits, and another one was the fact that the visits were time-consuming and required logistical planning. Overall, the professionals were positive about the home-visit program in that they felt that they were able to give the families what they needed and to have discussions on sensitive issues. They also appreciated the fact that different professions collaborated in order to reach the same goal.

**Conclusions:**

This study showed that the health, social, and dental professionals enjoyed working with the home-visit program and that they encountered positive feelings among the parents regarding the collaborative visits being conducted within the home, where the families felt safe and relaxed. The professionals expressed that the home visits, despite the extended time they required and the logistical challenges involved, created a deeper collaboration between the professionals.

## Introduction

The goal of the Swedish child healthcare (CHC) system is to reach all children with health-promotive actions from when the child is born until they reach school age [1]. Since the Swedish CHC works towards equal health opportunities for all children, an extended home-visit program, termed Grow Safely, seemed suitable for the county of Scania to engage in [2]. The purpose of this study was to evaluate the experiences that health, social, and dental professionals have had during their participation within this program. Similar collaborative programs involving different professions have been taking place at different places all over the world, for example, in the UK [3], the US [4–9], Australia [10], Switzerland [11], and Norway [12], to mention a few. However, to the best of our knowledge, the current study is the only one to have a geographical spread including both rural and urban areas and with CHC nurses, midwives, social workers, and dental assistants being involved in the same program. It is therefore important to evaluate Grow Safely not only from the perspective of the families [13] but also from the perspective of the professionals working within the program, since we want to highlight the collaboration between the professionals working within such a program.

According to laws within social services and healthcare in Sweden, healthcare settings and social services are obliged to collaborate with different professions [14, 15]. There is recurrent encouragement for health and social professionals to collaborate in preventive care for both families in general and families at risk of maltreatment [16]. However, collaboration between these professionals could be more difficult than imagined; it requires resources and it is often not that easy for professionals from different organizations [17]. Nevertheless, such collaboration is considered required for creating a holistic view on the matter that is targeted and especially with regard to families in need of support [18]. An earlier study indicates that interprofessional collaboration is essential in providing quality healthcare in today’s complex world and managing the challenges that healthcare systems face worldwide, and also that patients are in need of knowledge and skills that can only be found in a wide range of different healthcare professions [19]. In particular when there is a need to optimize newborn and maternal care, we need to draw strength from different kinds of health providers [19]. Interdisciplinary collaboration within maternal child health is needed at the local, regional, national, and international level, and there is a need to always keep the health and wellbeing of the mother and baby central in any decision making and practice [19]. However, since collaboration is difficult and challenging, research focusing on collaboration in regard to home visiting is of importance.

Nurturing care needs to be established and safeguarded through programs and services focusing on the support and provision of care for small children and aiming at enabling children to develop their full potential [20, 21]. This care should be offered through services and programs that collaborate in order to provide support to families according to their needs [20]. A holistic approach is put forward in this framework, where different sectors should be involved in creating cross-sectoral interventions, through using already existing platforms. The healthcare sector is considered a key strategic actor with the highest reach in interacting with families during the children’s early years [20, 21].

An earlier extended home-visit program, the Rinkeby project, focused on a disadvantaged area in Stockholm, Sweden, beginning in 2013, and the project has shown several benefits, such as increased parental confidence and knowledge of societal services and local resources for their families [22], as well as decreased caries frequency and improved tooth brushing [23]. Although this project has had some benefits, its focus has been on only one neighborhood in one city in Sweden and with only two professions involved.

The current study is part of the evaluation research that targets the Grow Safely program in the whole county of Scania in the south of Sweden, which has a population of around 1.3 million people [2]. Grow Safely, which was to some extent inspired by the Rinkeby project, started in 2019. One of the purposes of Grow Safely was to develop an interprofessional collaboration between CHC nurses, midwives, social professionals, and dental assistants through extended home visiting for first-time parents. Each home visit (six visits altogether for each family selected to participate in the program) was conducted by one CHC nurse together with either a midwife, a social worker, or a dental assistant. The long-term objective of the support provided by these home visits is to better contribute to easily accessible care and equal health for the population in the county [2].

Since the Swedish CHC centers mainly work independently of other professions, there is a great need to explore the health professionals’ experiences of working closely with other professions within an extended home-visit program and how such collaboration could benefit the professions. The aim of the current study is to illuminate the experiences that health, social, and dental professionals have had during their work within Grow Safely.

Research question: How did the professionals experience working within the program Grow Safely and how could they benefit from it?

## Methods

### Context

This descriptive qualitative study took place from early to late 2021 and interviews were carried out with health, social, and dental professionals working within CHC centers that had participated in the Grow Safely program in the county of Scania.

### Participants and data collection

The participants were recruited by emails sent out to all participating CHC centers. Together with the invitation for interviews, a written information sheet was sent to all staff explaining the aim of this study and that participation in the interviews was voluntary.

The first and the second author reached out to 22 CHC centers and requested participation in interviews. Five pediatric nurses, one district nurse, one midwife, one dental assistant, and five social workers replied and took part in the interviews, and they came from eight different CHC centers. In spite of reaching out to the 22 different CHC centers, only 13 professionals agreed to participate in the interviews and the sampling was therefore considered a convenience sampling. The professionals that agreed to participate were from the whole county and from both rural and urban areas. Due to the pandemic restrictions, the interviews took place online via Zoom. Before the interviews were conducted, each informant filled in a consent form agreeing to participation. Eight interviews were conducted by the first author and five interviews by the second author, the interviews being divided between the authors due to convenience and in the course of the informants’ consent to participate. Both authors have professional experience within qualitative research; the first author also has prior working experience from the field of child healthcare nursing and the second author has prior experience from the field of social work, which could give some pre-understanding of the research field.

The interviews focused on how the informants had experienced the urban and rural home visiting and the collaboration between the staff in the program. Furthermore, interviewees were asked about their views on what factors contribute to a functioning collaboration (see the interview guide, Table 1). Based on informed consent, the interviews were recorded on Zoom and transcribed shortly afterwards by the first and second author. The interviews lasted between 25 and 76 min.

**Table 1:** Interview guide

What was your overall experience of the home visiting? Can you tell us about a positive example of a home visit you conducted? Can you tell us about a negative example of a home visit you conducted? How has the collaboration in the team worked with respect to deciding the content of the visits? How have you experienced the coaching of the teamwork during the home visiting? How has your team functioned?	

### Analysis

When all 13 interviews were conducted, the interview data was coded by the first author. Then the coded material was read by the second author, who also gave feedback on it. The data was analyzed based on Burnard’s approach to content analysis [24]. Open coding was done by the first author and these codes were grouped into sub-categories. Then the material was searched for overlapping sub-categories before it was reduced to fewer sub-categories and categories. After clarifying the meaning of each sub-category, they were grouped into categories. For an example of the steps of the analysis, from meaning unit to code to sub-category, see Table 2, where the cells under “meaning unit” are examples from the interviews used to illustrate how the material was coded.

**Table 2:** Analysis process showing meaning unit, code, and sub-category

Meaning unit	Code	Sub-category
We could sense that the families are very happy and content when we visit them and that they feel safe (A11)	Sense that the families are content and feel safe	Feelings of satisfaction when working within the program
The visits are always planned with the aim of being flexible and seeing where the family is at that moment and to be able to sense what is important for them (A5)	Plan the visits and focus on where the family is and what is important for them	How the visits were prepared and conducted
*…the visits with interpreters have been challenging and it’s difficult for a translator through phone to discern the different roles in the visits and to be able to interpret […] correctly, and even if an interpreter is present during the visits, there are then more people during the visits and [it’s] quite hard when all have to communicate together (A1)*	There have been challenges with the interpreter participating over the phone and for the interpreter to be able to discern the information	Challenging aspects of the visits

### Ethical considerations

The interviews were carried out in line with the ethical code and guidelines in the Declaration of Helsinki [25]. The informants received both written and oral information about the study and about the fact that the interviews were voluntary and could be interrupted whenever they wanted. We only reached out to those CHC centers that answered the emails that were sent out with information about the study. All participating respondents in our study were provided with anonymity. In order not to reveal the identity of the professionals, the data was pseudonymized and their name and position, which would directly identify them, were replaced by a code (i.e., A1-13). As a result, the interviews were more open than they might have been otherwise, and both pros and cons of the project were touched upon by the respondents. No ethical application was carried out due to the informants not being considered a vulnerable group of people, and as no sensitive subjects were discussed and no sensitive personal data was collected [26]. However, the present study is a sub-study in the larger Grow Safely program, which has ethical approvals from the Swedish Ethical Review Authority (Reg. no. FO 4.3–2018 and Reg. no. 2019–03266).

## Results

The results are divided into three categories focusing on the perspectives of the professionals: (i) The value of home visiting from the perspective of parents and health professionals, (ii) Organizational aspects of working within the program, and (iii) How collaboration could benefit the families.

### The value of home visiting from the perspective of parents and health professionals

During the interviews, health, social, and dental professionals expressed what they as professionals saw among the parents receiving the program and how much the parents appreciated the fact that the visits took place in their own home. The professionals also expressed how satisfying it felt working within this program and how they connected deeper with the parents through the visits. This category contains two sub-categories: *The importance of being at home* and *Feelings of satisfaction when working within the program*.

#### The importance of being at home

The health, social, and dental professionals expressed that they perceived that the vast majority of parents were really positive about and grateful for the intervention they received and very much appreciated getting home visits and not having to go elsewhere for these meetings. They also expressed that they could notice the parents being thankful for receiving breastfeeding support during the visits. One social worker expressed her view like this:

“*Parents are safer in their own home and it creates a better balance between the professionals and the parents.”* (A11).

One of the nurses mentioned that the parents she met appreciated it most when they had conversations and discussions about important subjects, and one of the social workers said that the parents felt very happy with the home visits and that they felt safe during these meetings since they took place in the family’s own home.

#### Feelings of satisfaction when working within the program

Health, social, and dental professionals expressed feelings of being warmly welcomed by the families at the home visits and they were very happy about being part of this program. Furthermore, the midwife expressed that she felt like she got a deeper connection with the women during these home visits and that this felt very satisfying for her as a midwife. One of the pediatric nurses described it like this:

“*The home visits really make the contact with the whole family much better, and it feels fantastic to be able to give this program to these families.*” (A5).

One social worker expressed the excitement she felt when visiting first-time parents like this, since she normally had regular contacts every week with her clients at the CHC center. A sense of satisfaction and of being privileged to be able to work with these extended home visits, was something that most of the professionals expressed, and there was a desire that everyone who works in similar contexts should be able to carry out such home visits.

Further on, one social professional said that it felt very good to be able to have deep conversations with the parents and when the parents, after the visit, texted her and expressed their gratitude for it. Another social professional talked about a highly educated couple that they had met during the home visits, describing how, at the beginning, the conversations with the couple were very shallow and short, but adding that after a while the mother told them she had had feelings of inadequacy, and that seeing her open up felt very good for the professionals.

### Organizational aspects of working within the program

Under the category of organizational aspects, the professionals recounted how they prepared and conducted the visits, and how the discussions went regarding who should be prioritized for the visits, what challenging aspects they encountered, as well as the restrictions during the pandemic that modified the program. The sub-categories are: *How the visits were prepared and conducted, Prioritizing the program, Challenging aspects of the visits*, and *Restrictions during the pandemic that modified the program.*

#### How the visits were prepared and conducted

Several of the professionals stated that they based all visits on the manual that was created for this program, but that they were flexible and adapted their visits to the needs of each family. Others said that the manual helped and supported them during the visits, especially when they were new to this task. The structure of each visit depended on how well the professionals knew each other from before. One of the pediatric nurses expressed this as follows:

“*The first visit made by the midwife and a nurse is often shared equally by both, but the other visits are more often led by the nurse, since she often knows the families best.*” (A2).

One social worker said that they started each visit by connecting to the parents and listening to how they felt about and dealt with the situation of being new parents, and then they introduced themselves and explained what kind of roles they had. At the beginning of the program, they often decided before the meetings who should say what, but after a while they became more comfortable and could get into each other’s parts of the visits. Some subjects, such as relationships and parenting, were often led by the social worker, and other subjects, such as breastfeeding and health topics, were led by the nurse. But the more the professionals became comfortable and acquainted with each other, the more they could interrupt each other and not have to stick to their own professional role. The dental assistant said that even if her main focus was the teeth and dental health, she was very attentive to whether there were bigger problems and challenges that the family had, and that she would then withdraw somewhat from giving dental information and let the family direct the conversation to what was important for them.

#### Prioritizing the program

The professionals discussed which families should be prioritized for the program since it was not possible to include everyone. One nurse said that it felt good for her that they were able to offer the program to all first-time mothers at the specific CHC center where she worked, and that they did not have to decide whom to give and not to give the program to. One social worker from another CHC center said:

“*It feels like we have a lot of well-functioning parents in this area and they are not really in need of all these visits that we could give them.*” (A7).

One nurse agreed with this statement and said that it felt like a waste of their time to give all six home visits to well-functioning parents. The same nurse said that she would have liked to be able to give this program even to parents who were not first-time parents but who had an indicated need for the intervention. She also mentioned that some felt that too many home visits were made by the social workers, particularly when there was no special need for it, and that the home visit by the dental assistant was not really needed since the parents got similar information at a dental visit around the same time. Moreover, there were some CHC centers that had a wider area geographically and were not able to give the program to parents living in the outer parts of the area, and that was perceived as negative by this nurse.

#### Challenging aspects of the visits

Mostly the professionals were very pleased with the home visits, but there were a few aspects that were negative or challenging for the professionals involved in the program. Several of the professionals mentioned that the time aspect was challenging, since the program with its home visits required more time and more preparation than their regular work and since it was sometimes difficult, logistically, to book and arrange the visits, as it had to work for two different professionals, with different calendars. Another challenging aspect was that some visits required an interpreter, and one nurse described it like this:

“*In general, the visits with interpreters have been challenging and it’s difficult for a translator through phone to discern the different roles in the visits and to be able to interpret […] correctly, and even if an interpreter is present [in the room] during the visits, there are then more people during the visits and [it’s] quite hard when all have to communicate together.”* (A1).

Yet another challenge for the professionals to deal with was when, on some occasions, there were more family members present during the visits than the parents and their child, and when these, for example grandparents, sometimes intervened in the dialogue and took up a lot of space during the conversations. It was also reported that sometimes some family members came and went during the visits, which could create a bit of a disturbance.

#### Restrictions during the pandemic that modified the program

Shortly after the start of Grow Safely, the Covid-19 pandemic broke out, which affected the organizational aspects of the program. Some of the home visits could not be conducted because of restrictions. Instead, some of the meetings had to take place at the CHC center, and one social worker said this:

*“We had to conduct the visit in a corona-specific room at the CHC center instead of doing a home visit.”* (A7).

There were restrictions at the CHC centers as well, all staff and the families attending having to keep their distance from each other, and the professionals talked about having a flexible attitude concerning where and how to conduct the visits, while always focusing on what was in the best interest of the families. Some of the professionals talked about having taken outdoor walks with the families in order to meet them face to face. The effect of the pandemic and its restrictions differed between the professionals involved in the program. Some of the social workers were working from home and were not allowed to physically meet families, but despite restrictions some social workers were allowed to visit the families in the current intervention. There were also different recommendations regarding the wearing of face masks; for some of the professionals it was recommended that they wear face masks during all encounters and for some only during patient-close contacts. During the home visits, however, they agreed on always wearing masks, in order to approach all the families in the same way. Although they had to follow the restrictions, one of the social workers mentioned that something is missed when meeting families at the CHC center, instead of meeting them in their homes and seeing them in their own environment.

### How collaboration could benefit the families

The professionals talked about how they, through the collaboration within the program, were able to give the families what they needed and to discuss sensitive and difficult issues or subjects, and about how they were collaborating in their aim to reach and support the families. The following sub-categories were formed: *Being able to give the family what they need, Being able to talk about sensitive issues together*, and *Different professions working towards the same goal.*

#### Being able to give the family what they need

One of the social workers talked about the great privilege of being able to visit families before a problem or challenge arose, and being able to support and help families with what was needed together with the nurses. Another social worker talked about how she listened to where the family was at the time of the home visit, focusing on how they could help the family. And one social worker recounted how they supported parents with babies that had colic pain and how they as social workers could complement the nurses with their advice. She described it like this:

*“When we visit parents with a baby that has colic pain, we bring a doll and then demonstrate how to do baby massage to relieve the pain.” (*A11).

One nurse said that she thought that the aim of the home visits was to establish an early contact and to build trust between the different professionals and the parents. She also said that being able to give information early to the family and help them create good routines in their daily life, was beneficial not only for the child and the family but also for society as a whole.

#### Being able to talk about sensitive issues together

One of the nurses talked about the challenge of the 10-month home visit, where they were supposed to bring up the subject of child abuse and inform about this. The social workers had more experience of dealing with this matter compared to the CHC nurses, and there were discussions about whether the information could be given more naturally to all parents, without distinguishing those who were considered to need it most. For the pediatric nurses, the subject of child abuse was more challenging and sensitive than for the social professionals.

This was confirmed by several of the social professionals, and one social worker mentioned that it is important for the professionals to remember their own situation, that is, how they handle their own children, and how difficult this could sometimes be, something that could create humbleness in the professionals when communicating about this sensitive issue. Another social worker described how they handled situations when the baby cried and they had to talk about how these kinds of situations could trigger aggression in parents. She described it like this:

*“We discuss with the parents that baby cries could release different kinds of feelings in the parents and that they need to know where to seek help and support if they feel like they can’t handle it safely.”* (A11).

The same social worker related how she informed about how to set boundaries for the child and encouraged parents to be bold and tell the child when they are not allowed to do or touch something, for example. Another sensitive issue was how to present to the parents that the social workers came from the social services, which was sensitive to some parents and needed to be communicated along with trust building in order to create trust in the social welfare system.

#### Different professions working towards the same goal

One nurse talked about the importance of connecting families to the social services at an early stage of the babies’ lives, and through Grow Safely this could be done in a natural way. One social worker said that she believed that even if CHC nurses have an important role in the interaction with the families, social workers could also contribute positively to the families’ wellbeing and the role that parenting has for the parents. Another social worker stated that earlier collaboration played a role in the good interaction between the professionals in Grow Safely, expressing it like this:

“*We have worked together before within the family CHC center, so therefore it was easier to collaborate and work with the families together.”* (A9).

One of the nurses described the first visit together with the midwife and how important that visit was for reporting the family between the maternal healthcare and the CHC, stating that a midwife is more capable of answering questions related to pregnancy and different feelings and issues after the delivery. A good collaboration between the maternal healthcare and the CHC is important when encountering women with, for example, post-partum depression. After that first visit, a social worker joined the nurse and the nurse was thus able to connect the family to this person. One social worker expressed that it was much less dramatic to be presented as social workers by the CHC nurse compared to coming by themselves. The professionals also mentioned that they could learn from each other during the visits and thereby improve their way of communicating with and supporting the families.

## Discussion

The results revealed that the health, social, and dental professionals had feelings of satisfaction while working with the home-visit program and that they encountered positive feelings among the parents receiving it. The professionals followed a manual for the home visits but expressed the need for flexibility in order to see and meet the parents where they were. They discussed how the program could, if needed, be prioritized to fewer families with more needs and how the recent pandemic affected the visits. The negative part was the difficulty of handling interpretation while doing some of the visits and the fact that the visits required more time and more logistical planning than their regular work. There was an overall positive feeling that the program could, through collaboration, give the families what they needed and that it could open up for sensitive issues, as well as showing that different professions could work together with the families in order to reach the same goal of increasing the support and help for the parents who received the home visits.

### The value of home visiting from the perspective of parents and health professionals

A positive aspect of the home visits was the creation of deep conversations and the opening up about feelings that could otherwise be shameful to express. Parents interviewed in the same research project expressed how they felt safe and secure receiving the combined home visits instead of going down to the CHC center [13]. Research shows that the home is a place of security, where people can feel that they are able to control their situation and relax even when they experience challenges that seem to be beyond their control [27]. This could explain why the professionals sense and understand that the parents are more open and at ease during the meetings and dialogues in their own home environment, compared to when they are at the CHC center.

The health, social, and dental professionals saw positive effects for the parents receiving the program, not least because they were able to get the parents to talk more openly about their feelings and struggles regarding parenting, which is very important in order to increase the wellbeing of both children and their parents. There are studies demonstrating that parents who are able to be insightful of their children’s motives and behaviors during their interaction with them, are more likely to have securely attached children [28, 29]. It is therefore important that parents get the opportunity to open up about feelings and struggles that they face and through this open communication be strengthened in getting a better insight into their children’s behavior and improve their interaction with the child.

### Organizational aspects of working within the program

Organizational aspects, such as logistical planning, were mentioned as a negative facet of the experience of working within the program, something which is also shown in earlier research on interprofessional collaboration [17]. The overall experiences were positive, however, and the health, social, and dental professionals felt that the feedback they got from the parents was rewarding. This is an important part of the work, not least since being able to work in such a program as Grow Safely could attract more applicants to pediatric specialized nursing. There has for a while been a shortage of specialist pediatric nurses in Sweden and it is important to attract more nurses to the field of CHC in the future in order for the CHC centers to be able to carry out the logistical planning and work that a home-visiting program such as the one described in this study entails. Some of the professionals mentioned that the work with the extended home-visit program was time-consuming, which indicates that the heads of the CHC centers and the social welfare centers need to prioritize this task and provide the needed resources and money for it, in order for these kinds of projects to last for longer than a short period.

The discussion on prioritization, that is, which families should be included in the program, was part of the interviews, and now the Swedish government has decided that efforts and money will be invested in extended home-visit programs aimed at lower socio-economic areas in Sweden [30]. This is in line with the WHO Commission on Social Determinants of Health, stating the importance of working for health among all in a country and trying to level out the imbalance that could be seen in regard to the health among people [31]. It could still be discussed whether there could not also be a need for such a program for the families living in higher socio-economic areas, as they face challenges and difficulties as well, if sometimes of a different kind. But because of restricted resources, it seems as if this kind of program needs to be prioritized to the groups of families that need it most.

### How collaboration could benefit the families

As mentioned before, collaboration between different professions within social work and healthcare is considered required for creating a holistic view of the matter that is targeted and especially with regard to families in need of support [18]. This was evident in the interviews, where the health, social, and dental professionals expressed how the collaboration with different professions improved their encounters with the families, as well as the information and the support given to the families during the home visits. One benefit that joint home visiting could have is saving the parents from repeating the same information to different health professionals and thereby avoiding the risk of conflicting advice [3]. In the current study, the health and social workers described how they helped each other with the conversations with the families and how they could complement each other through the home visiting, as well as learning from each other’s way of interacting with the families during the visits. Not only the visited family but also other families could benefit from the fact that the professionals develop themselves during the collaboration with the other professions.

In the present study, it was revealed that the program lowered the barrier for turning to the social services and improved the reputation that social welfare has among some people in Sweden. A correct and true view of the social services is very important, since one of their goals is to support parents in their parenting at an early stage [32]. Nevertheless, a collaboration between CHC nurses and social workers could address some challenges in connection with their assessments and decision-making processes that are important to consider. For example, an American study by Williams et al. [33] found that CHC nurses and social workers determined to submit a report to social welfare based on different concerns, which highlights that poor communication between social workers and CHC nurses could have detrimental impacts on families involved in their care [33]. Effective collaboration requires knowledge of the functioning and processes of the other organization, mutual trust, and shared commitment to a common goal [34, 35]. It is therefore important to evaluate how diverse teams collaborate when working with families. Our study indicates that, in the case of Grow Safely, the professionals have a well-functioning collaboration. The quotes from the interviews suggest that the professionals can sometimes carry out the same tasks, despite their professional differences. That is to say, they can, for example, comment on things that are outside their respective professional authority and thereby develop a deeper form of collaboration [18].

The professionals could also see that through collaboration they were able to, at an early stage, support families in need of support in different ways, which is in accordance with earlier research [18]. Moreover, they were able to help the families get closer to the different health and social workers involved in the program and thereby get closer to the support needed in different situations. This is also confirmed by earlier studies and reports [20, 21] mentioning that nurturing care needs to be established and safeguarded through programs and services focusing on the support and provision of care for small children and aiming at enabling children to develop to their full potential. Another study, by Brodie and Knight [36], showed that collaborative home visiting could benefit health professionals regarding the communication of their concerns about families to other healthcare professionals, such as GPs, who rarely see the families in their homes. Through such programs as Grow Safely, the health and social workers could collaborate with GPs and other relevant professionals around issues relevant for reaching better and more equal health and care among children in Sweden.

### Limitations

This program started shortly before the Covid-19 pandemic hit the globe, which of course affected the way research could be conducted. The contacts with the professionals at each CHC center were established through either physical visits or digital meetings. The interviews were held through Zoom, which worked out well; however, we are not able to say how the flow in the interviews would have been if they had been conducted through physical meetings instead. Recent research shows that Zoom interviews have cost benefits as well as the benefit of being able to reach more remote interviewees, but that there could be disadvantages, too, in not seeing the person in the flesh, especially when sensitive issues are discussed [37]. However, the current study cannot be considered to deal with a sensitive subject and therefore we hope that the technique did not interfere with the results. The first and the second author conducted the interviews separately and both are experienced researchers within qualitative method. Both authors also have pre-understanding from working within CHC and social welfare, respectively, and this is something we have been aware of and discussed among us. The interviews were done in Swedish and then translated into English while writing the results, and we have tried to ensure that no important nuances or other details were lost in translation. The sample was convenience sampling and the health, social, and dental professionals that were willing to do so participated in the interviews. There were several nurses and social workers but only one midwife and one dental assistant, which is a limitation. The convenience sampling could also have led to more professionals with a positive view of the Grow Safely program being interviewed and this needs to be considered while interpreting the results in the current study.

To ensure credibility, the coded material was read independently by both authors and the coding was discussed and compared between the first and the second author until we reached agreement on the final version of the analysis [38]. Credibility could also be seen in the extent to which judgements about similarities and differences were consistent over the interview process, and in the fact that an open dialogue between the authors took place during the research process in regard to decisions about the focus of the study, the selection of context, the participants, and the approach to gathering data [39]. Dependability was ensured by the process of data analysis and by allowing the steps of the chosen method to be followed closely [39]. As to transferability, it could be related to the decision-making process of those practitioners or researchers that are seeking to transfer the study findings to their own settings [38], but our findings could be limited in their transferability since only 13 health and social workers were interviewed out of 165 health and social workers working with Grow Safely within the Region of Scania.

## Conclusion

The current study showed that the health, social, and dental professionals had a good impression of the home-visit program. The professionals expressed satisfaction with working within the extended home-visit program, largely because they were able to see parents open up in the dialogues, which they believed was to a great extent due to the parents feeling secure in their home environment. They discussed how the program could be prioritized to fewer families with more needs, if necessary, and how the recent pandemic affected the visits. Although there were some logistical challenges within the collaboration, the professionals expressed that the home visits created a deeper collaboration between the health, social, and dental professionals.

## Data Availability

The datasets generated or analyzed during the current study are available from the corresponding author on reasonable request.

## List of Abbreviations

CHC: Child healthcare

## References

[1] Rikshandboken Barnhälsovården, [http://www.rikshandboken-bhv.se/].

[2] Mangrio E, Hellström L, Nilsson E-L, Ivert A-K. An Extended Home visit Programme within the swedish child Healthcare System for First-Time parents in scania, Sweden: a study protocol. Front Public Health 2021, 9. 10.3389/fpubh.2021.537468 PMC7900174 33634063

[3] Olander EK, Aquino MRJ, Bryar R. Three perspectives on the co-location of maternity services: qualitative interviews with mothers, midwives and health visitors. J Interprof Care 2020:1–9. 10.1080/13561820.2020.1712338 32013629

[4] Rushton FE, Byrne WW, Darden PM, McLeigh J (2015). Enhancing child safety and well-being through pediatric group well-child care and home visitation: the Well Baby Plus Program. Child Abuse Negl.

[5] Finello KM, Terteryan A, Riewerts RJ (2016). Home visiting programs: what the primary care clinician should know. Curr Probl Pediatr Adolesc Health Care.

[6] Duffee JH, Mendelsohn AL, Kuo AA, Legano LA, Earls MF, Chilton LA, Flanagan PJ, Dilley KJ, Green AE, Gutierrez JR. Early childhood home visiting. Pediatrics 2017, 140(3). 10.1542/peds.2017-2150 28847981

[7] Gomby DS. Home visitation in 2005: Outcomes for children and parents. In: Invest in Kids working paper; 2005.

[8] Duggan A, Portilla XA, Filene JH, Crowne SS, Hill CJ, Lee H, Knox V. Implementation of evidence-based early Childhood Home Visiting: results from the mother and infant home visiting program evaluation. OPRE Report 2018-76A. Office of Planning Research and Evaluation 2018.

[9] Dodge KA, Goodman WB, Bai Y, Murphy RA, O’Donnell K (2021). Maximizing the return on investment in early childhood home visiting through enhanced eligibility screening. Child Abuse Negl.

[10] McDonald M, Moore T, Goldfeld S. Sustained nurse home visiting for families and children. 2016.

[11] Kurth E, Krähenbühl K, Eicher M, Rodmann S, Fölmli L, Conzelmann C, Zemp E (2016). Safe start at home: what parents of newborns need after early discharge from hospital–a focus group study. BMC Health Serv Res.

[12] Dahlberg U, Haugan G, Aune I (2016). Women’s experiences of home visits by midwives in the early postnatal period. Midwifery.

[13] Forss KS, Mangrio E, Hellström L. Interprofessional Teamwork to Promote Health: First-Time Parents’ Experiences of a Combined Home Visit by Midwife and Child Health Care Nurse. Front Pead 2022, 10. 10.3389/fped.2022.717916 PMC8927075 35311059

[14] Sveriges Riksdag. : Socialtjänstlag (2001:453). In. Stockholm; 2001.

[15] Sveriges Riksdag. : Hälso- och sjukvårdslag (2017:30). In. Stockholm; 2017.

[16] Hessle S (2003). Positioneringar i den sociala barnavårdens kontext. Fokus på barn, familj och nätverk-metodutveckling i den sociala barnavården.

[17] Boklund A (1995). Olikheter som berikar?: möjligheter och hinder i samarbetet mellan socialtjänstens äldre- och handikappomsorg, barnomsorg samt individ- och familjeomsorg.

[18] Hjortsjö M (2005). Med samarbete i sikte: Om samordnade insatser och samlokaliserade familjecentraler.

[19] Shamian J (2014). Interprofessional collaboration, the only way to save every woman and every child. The Lancet.

[20] World Health Organization. : Nurturing care for early childhood development-A framework for helping children survive and thrive to transform health and human potential. In; 2018.

[21] Britto PR, Lye SJ, Proulx K, Yousafzai AK, Matthews SG, Vaivada T, Perez-Escamilla R, Rao N, Ip P, Fernald LC (2017). Nurturing care: promoting early childhood development. The Lancet.

[22] Mekhail KT, Lindberg L, Burström B, Marttila A (2019). Strengthening resilience through an extended postnatal home visiting program in a multicultural suburb in Sweden: fathers striving for stability. BMC Public Health.

[23] Brännemo I, Dahllöf G, Cunha Soares F, Tsilingaridis G (2021). Impact of an extended postnatal home visiting programme on oral health among children in a disadvantaged area of Stockholm, Sweden. Acta Paediatr.

[24] Burnard P, Gill P, Stewart K, Treasure E, Chadwick B (2008). Analysing and presenting qualitative data. Br Dent J.

[25] WMA Declaration of Helsinki. : Ethical Principles for Medical Research Involving Human Subjects. In, vol. 2018. 19886379

[26] Swedish Government. : Lag (2003:460) om etikprövning av forskning som avser människor. In. Stockholm; 2003.

[27] Dupuis A, Thorns DC (1998). Home, home ownership and the search for ontological security. Sociol Rev.

[28] Koren-Karie N, Oppenheim D, Dolev S, Sher E, Etzion-Carasso A (2002). Mothers’ insightfulness regarding their infants’ internal experience: relations with maternal sensitivity and infant attachment. Dev Psychol.

[29] Oppenheim D, Koren-Karie N, Sagi A (2001). Mothers’ empathic understanding of their preschoolers’ internal experience: relations with early attachment. Int J Behav Dev.

[30] Uppdrag att stödja. och stimulera barnhälsovårdens förebyggande arbete med hembesöksprogram (In Swedish), [https://www.regeringen.se/regeringsuppdrag/2023/05/uppdrag-att-stodja-och-stimulera-barnhalsovardens-forebyggande-arbete-med-hembesoksprogram/].

[31] World Health Organization. Closing the gap in a generation: health equity through action on the social determinants of health: commission on Social Determinants of Health final report. World Health Organization; 2008. 10.1016/S0140-6736(08)61690-6 18994664

[32] Socialtjänstens öppna verksamheter. för barn och unga [https://www.socialstyrelsen.se/globalassets/sharepoint-dokument/artikelkatalog/ovrigt/2009-126-145_2009126145_tryckfil.pdf].

[33] Williams VN, Ayele R, Shimasaki S, Tung GJ, Olds D (2019). Risk assessment practices among home visiting nurses and child protection caseworkers in Colorado, United States: a qualitative investigation. Health Soc Care Commun.

[34] Corbin JH, Jones J, Barry MM (2018). What makes intersectoral partnerships for health promotion work? A review of the international literature. Health Promot Int.

[35] Lee J, Palekar US, Qualls W (2011). Supply chain efficiency and security: coordination for collaborative investment in technology. Eur J Oper Res.

[36] Brodie T, Knight S (2014). The benefits of multidisciplinary safeguarding meetings. Br J Gen Pract.

[37] Thunberg S, Arnell L. Pioneering the use of technologies in qualitative research–A research review of the use of digital interviews. Int J Soc Res Methodol 2021:1–12.

[38] Lincoln YSGE. Naturalistic Inquiry. SAGE Publications; 1985.

[39] Graneheim UH, Lundman B (2004). Qualitative content analysis in nursing research: concepts, procedures and measures to achieve trustworthiness. Nurse Educ Today.

